# Biological Effects of Orthodontic Tooth Movement on the Periodontium in Regenerated Bone Defects: A Scoping Review

**DOI:** 10.3390/dj12030050

**Published:** 2024-02-26

**Authors:** Alessio Verdecchia, Carlota Suárez-Fernández, Andrea Miquel, Giulia Bardini, Enrico Spinas

**Affiliations:** 1 Department of Surgery and Medical-Surgical Specialities, School of Medicine and Health Sciences, University of Oviedo, 33003 Oviedo, Spain; suarezcarlota@uniovi.es; 2 Department of Surgical Sciences, School of Periodontology and Implantology, Mississippi Institution, 28010 Madrid, Spain; miquel.c.andrea@gmail.com; 3 Department of Surgical Sciences, Division of Conservative Dentistry and Endodontics, University of Cagliari, 09124 Cagliari, Italy; giulia.bardini@unica.it; 4 Department of Surgical Sciences, Postgraduate School in Orthodontics, University of Cagliari, 09124 Cagliari, Italy

**Keywords:** orthodontic tooth movement, bone defect, bone graft, periodontal effects

## Abstract

The aim of this scoping review is to analyse the biological effects of the orthodontic tooth movement (OTM) in areas with bone defects that are undergoing regeneration using different types of regenerative materials and techniques. The electronic research was performed on four databases as follows: PubMed, Scopus, EMBASE, and Web of Science. Data were extracted according to publication information, study design, sample characteristics, parameters of OTM, biological repercussions on the periodontium complex, methods of analysis, and conclusions. A total of thirty studies were included in the final review. In twenty-two studies, the most widely adopted grafting materials were alloplastics. In most studies, the orthodontic force used was 10 or 100 g, and the timing of application ranged from immediate to 6 months after grafting surgery. Twenty-four studies showed an increase in osteogenesis; in five studies, the clinical attachment level (CAL) increased; in five others, the probing pocket depth (PPD) decreased; in sixteen studies, there was root resorption of a different magnitude. Though the effects of OTM on the periodontium in the grafted areas were positive, the outcomes should be interpreted with caution as future preclinical and clinical studies are needed to extrapolate more valid conclusions.

## 1. Introduction

Bone grafts and substitute materials play a crucial role in regenerating missing hard tissue structures, providing a structural framework for osteo-regenerative processes [1]. Autologous bone, whether used alone or in combination with bone substitute materials, and bone substitute materials on their own are well-established materials [2]. These substitute materials can be categorised into those of natural origins such as autografts, allografts, and xenografts, or alloplastic materials. While autografts are considered the gold standard for treating bone defects, synthetic grafts remain the most common choice [3].

Orthodontic treatment induces changes in the alveolar bone, affecting the characteristics of the bone differently across various regions of the mouth. Specifically, orthodontic treatment leads to a reduction in bone thickness, particularly on the palatal side and in the incisor region [4]. It also produces changes at the gingival tissue level, including gingival recession [5]. Root resorption is another side effect of orthodontic movements, especially when continuous and heavy forces or a higher amount of apical movement over a long duration are applied [6].

Teeth can also be moved through bone grafts despite there being a side effect of occasional slight root resorption. However, it is essential to carry out these movements when the bone is adequately prepared [3]. Previous studies suggest that allografts and β-tricalcium phosphate influence bone homeostasis, resulting in a deceleration of orthodontic tooth movement in regenerated sites [7]. The aim of this study is to analyse the biological effects of orthodontic tooth movement on the periodontium in areas with bone defects that are undergoing regeneration.

## 2. Materials and Methods

### 2.1. Protocol and Registration

This scoping review was conducted following the Preferred Reporting Items for Systematic Reviews and Meta Analyses Extension for Scoping Reviews (PRISMA-ScR) guidelines [8].

### 2.2. Information Sources and Search Strategy

A comprehensive search strategy was designed, and a thorough computer-based search was performed on 10 November 2023, using the electronic databases as follows: PubMed, Scopus, EMBASE, and Web of Science. An electronic search of the grey literature was made through Opengrey. The strategies designed for each database are shown in Table 1. No limitation of language or publication date was applied in the literature search.

### 2.3. Study Selection and Eligibility Criteria

The electronic search was independently conducted by two investigators (AV and CSF), screening titles and abstracts in parallel to evaluate the studies for eligibility. In case of missing information, full-text reading was necessary for a final decision. A third author (ES) discussed and resolved any discrepancies between the two authors. No language or time limit was applied to the search strategy. The inclusion criteria for the selection of articles had to accomplish the following characteristics: (1) human and animal subjects, (2) orthodontic movement across bone defects repaired with synthetic or natural material grafts, (3) biological and/or biomechanic repercussions on the tooth and/or adjacent tissues to the orthodontic displacement, (4) and the amount of orthodontic tooth movement produced across the bone defects repaired. On the other hand, the exclusion criteria were as follows: (1) in vitro and ex vivo studies, (2) literature reviews, (3) and systematic reviews.

### 2.4. Data Extraction and Synthesis

Two different reviewers (A.V. and C.S.F.) unitedly determined which variables to extract and developed the data-charting form. The data were extracted independently by the same two reviewers. The data were extracted according to the following: publication information (authors of the study and year of publication); study design (type of study); sample characteristics (including species, age, gender, type of defect, defect size, and localization and material of regeneration); parameters of orthodontic tooth movement (including localization of orthodontic tooth movement, timing, direction, magnitude, as well as mode of the force application, total duration of orthodontic tooth movement, and amount of movement achieved); biological repercussions on the periodontium complex (including bone formation/resorption, clinical attachment level, root integrity/resorption, and probing pocket depth as a response to the orthodontic tooth movement); methods of analysis; and conclusions. The extracted outcomes were retrieved from the included studies and then summarised.

## 3. Results

### 3.1. Literature Search and Screening Process

**Figure 1:**
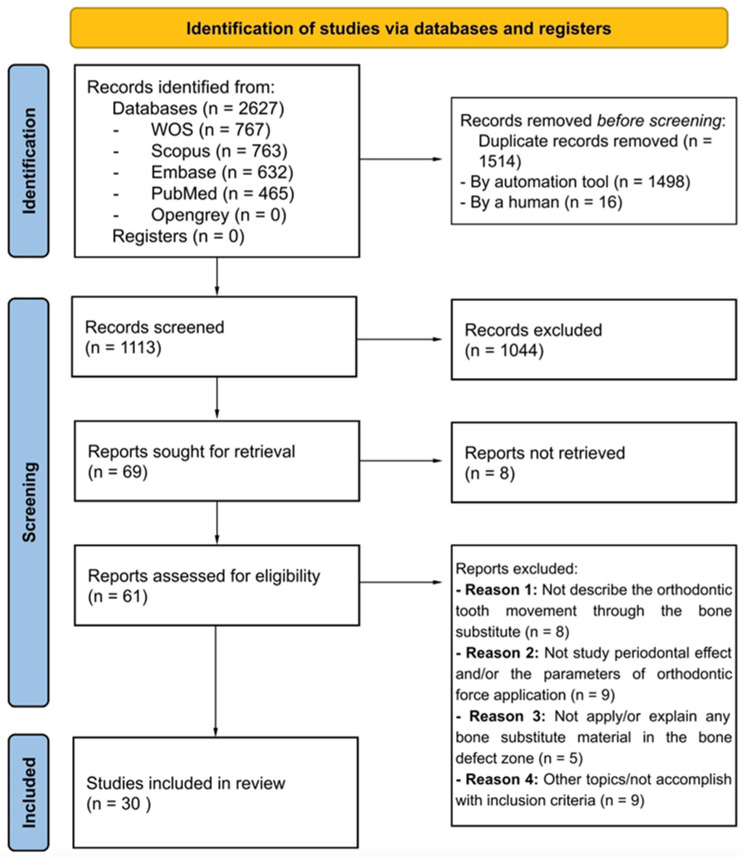
Flow chart showing the literature search and selection.

The search strategy identified 2627 publications from the PubMed (*n* = 465), Scopus (*n* = 763), EMBASE (*n* = 632), Web of Science (*n* = 767), and Opengrey (*n* = 0) databases. Following the removal of duplicated ones, 1113 records remained. After reading the title and abstract, an additional 1044 studies were excluded. Subsequently, the full text of 61 out of the remaining 69 studies was reviewed; 8 studies could not be retrieved. Among the studies whose full text was read, eight were excluded for not studying orthodontic tooth movement through bone substitutes, nine for not analysing the effects on the periodontium and/or describing the parameters of the applied orthodontic force, five for not mentioning and/or not explaining the type of material used to treat the bone defect, and nine for not addressing the topic under study and not meeting the inclusion criteria. In the end, 30 studies were included that met the inclusion criteria for this scoping review. The details of the literature search and selection procedure are shown in a flow chart in Figure 1.

### 3.2. Description of the Included Studies

Table 2 describes the main characteristics of the studies included in this scoping review. The studies were published between 1996 [9] and 2022 [10] and were mostly developed in China (*n* = 13) [11,12,13,14,15,16,17,18,19,20,21,22,23], which was followed by Japan (*n* = 3) [24,25,26], Germany (*n* = 3) [10,27,28], Israel (*n* = 2) [7,29], Korea (*n* = 2) [30,31], Italy (*n* = 1) [32], Brazil (*n* = 1) [33], Bangladesh (*n* = 1) [9], Sweden (*n* = 1) [34], Turkey (*n* = 1) [35], Egypt (*n* = 1) [36], and the United States of America (*n* = 1) [37].

#### 3.2.1. Study Design

Almost all the included studies (*n* = 27) were experimental studies. Additionally, five case reports [23,28,32,35,37] and only one observational one [15] were included in this scoping review.

#### 3.2.2. Sample Characteristics

The species used in the included works exhibited variability across the different studies. Dogs were employed in most experiments (*n* = 12) [9,21,24,25,30,34]. Seven studies incorporated humans as the subjects of analysis [15,23,28,32,35,36,37], while six studies involved rats [10,16,17,18,19,27], two studies used mice [7,29], two studies utilised rabbits [12,21], and the sample comprised minipigs in one study [33].

The total number of subjects across all species was 581, including 84 dogs (51 males, 15 females, 18 N/R), 62 humans (13 males, 19 females, 30 N/R), 261 rats (261 males), 98 mice (98 males), 70 rabbits (N/R), and 6 minipigs (6 males). Table 3 specifies the number of subjects by species, gender, and age.

#### 3.2.3. Type of Bone Defects

The nature of the defect varied among the selected studies. Sixteen studies addressed extraction socket grafts [7,9,11,12,14,16,17,18,22,24,25,28,29,30,33,34], seven focused on periodontal sockets [13,20,23,31,32,36,37], and an additional seven dealt with alveolar clefts [10,15,19,21,26,27,35]. Concerning their location, 21 articles examined defects in the upper jaw, 3 in the lower jaw [12,22,34], and 5 in both bones [14,20,28,31,33]. Only one study did not specify the location of the defect [36].

#### 3.2.4. Type of Graft Materials

In the included studies, a variety of graft materials were used, encompassing autografts, xenografts, allografts, alloplastic materials, and stem cells. A total of 17 studies used different types of materials to make comparisons. Out of the studies, 22 utilized alloplastic materials [7,9,10,11,12,14,16,17,18,20,21,22,24,25,26,27,28,31,33,35,36,37], 15 employed xenografts [10,11,13,14,16,17,18,23,27,29,30,31,32,33,34], 10 utilized autografts [7,9,10,12,15,19,21,25,26,27], 2 employed allografts [31,35], and 2 additionally incorporated the use of stem cells [21,22].

#### 3.2.5. Parameters of Orthodontic Tooth Movements

The characteristics related to the analysed parameters of orthodontic tooth movement are described in Table 4.

- Localization of orthodontic movement

The main location where tooth movement was examined was the maxilla (*n* = 30). In four studies, orthodontic movement in the mandible was also analysed simultaneously with the upper jaw [14,20,31,33]. Most studies used the canine as the anchoring unit (*n* = 12) and the premolar as the unit of movement (*n* = 10).

- Timing of orthodontic force application

According to the analysed studies, the time at which the orthodontic force is applied and tooth movement is initiated is quite variable and is within a rank from immediate to 6 months after surgical grafting treatment. In five studies, treatment was initiated immediately [12,13,30,31,36], after one week in one study [37], after two weeks in three studies [9,22,30], per month in as many as eleven studies [7,9,10,13,14,16,17,18,22,27,29], after a half month in one study [28], in six articles at two months [13,14,19,21,22,36], in five studies after three months [22,23,30,33,34], in two studies at four months [24,25], and in three studies at six months [20,26,35]. Five studies also studied orthodontic movement in multiple time lapses from two to four times [13,14,22,30,36]. In two articles, the exact time of the force application was not reported [11,15].

- Direction of orthodontic force

The force was applied in various directions depending on the orthodontic movement required to move the tooth into the grafted area. In most of the studies, the force presented a mesial direction, which was demonstrated in as many as 20 of them [7,10,11,12,14,15,16,17,18,19,22,24,25,26,27,29,30,32,33,35]. In four studies [9,21,26,34], the movement was realized in the distal direction, in two this was apically [23,37], and in three this was buccally [13,20,31]. In two studies, this was not reported [28,36].

- Magnitude of orthodontic force

The magnitude of the applied force based on the data analysed was highly variable, ranging from 10 g to 458.87 g (4.5 N). The majority preferred to apply a force of 100 g [19,20,24,25,26,30]. The magnitude of 10 g was also widely applied [7,16,18,29]. In four studies, these data were omitted [9,15,32,35].

- Mode of orthodontic force application

Various modalities were described for tooth movement to be achieved. In 23 studies, the method applied was through the use of a closed coil spring [7,9,10,11,12,13,14,15,16,17,18,19,21,22,24,25,27,28,29,30,31,33,34,35]. In three studies, this was realized by a continuous arch without further specification [15,20,37]. A further three studies used the segmented technique [23,32,36]. Only one study adopted the elastic chain [26].

- Total duration of orthodontic tooth movement

In the included articles, the total duration of orthodontic movement was also very heterogeneous, ranging from 5 days to 32 months. In animal studies, the analysis of the total duration of tooth movement ranged from 5 days to 6 months [7,9,10,12,16,17,18,19,21,24,25,27,29,30,33,34]. Meanwhile, in some studies performed on humans [23,28,32,35,36], the temporal rank, despite being very variable, exceeded 6 months and reached a maximum of 32 months.

#### 3.2.6. Biological Repercussions on the Periodontium Complex and Methods of Analysis

The characteristics related to the biological repercussions on the periodontium complex and the methods of analysis of orthodontic tooth movement are described in Table 5.

- Bone response

In as many as 24 studies, most of the results showed that there was an increase in bone formation compared with that of the control group after an initial phase of bone turnover in the graft zone. Among these, five compared various types of bone grafts with each other. In Ru’s three studies [16,17,18], synthetic bone resulted in higher bone regeneration than that observed for xenogenic bone graft substitutes. In Moehlhenrich’s 2021 study [27], xenogeneic and autologous bone substitutes exhibited higher bone formation than synthetic substitutes. Two studies converged in stating that conjugating bone marrow stromal cells with Beta tricalcium phosphate resulted in higher bone regeneration than that observed for Beta tricalcium phosphate when it was used alone [12,21]. Only one article [33] was explicit in stating that the process of bone regeneration and resorption were in balance. In three studies [10,22,28], no such information was reported, and one study [20] did not detail the bone-level response following grafting.

- Clinical attachment level (CAL)

Most studies did not report the effects of orthodontic movement on CAL. An increase in the clinical attachment level was observed in the area undergoing treatment in only five studies [9,23,32,36,37].

- Probing pocket depth (PPD)

Regarding probing pocket depth, the majority of the studies also did not mention this parameter. Five studies reported a decreased probing pocket depth [11,23,32,36,37], and only Lee’s study observed an increase in the probing pocket depth [31].

- Root integrity/resorption

From the results obtained, root resorption as an effect of orthodontic tooth movement in a bone graft zone was produced in nine studies, albeit with a different degree of magnitude. In seven articles, there was slight or partial root resorption [9,15,24,25,31,33,34], while in one the extent of resorption was more important [10]. In five articles, no root resorption was reported [12,26,28,35,37]. In three studies, it was stated that synthetic bone substitutes induced a lower degree of root resorption than that observed for xenografts. No such information was reported in 13 studies.

- Methods of analysis

The selected articles mainly used histological and histomorphometric analysis as the method of choice. Clinical examination was conducted in nine studies [11,14,15,28,31,32,35,36,37]. In eight studies, the conventional radiographic method was chosen [9,15,21,26,28,35,36,37]. For the three-dimensional study, the analysis was conducted through micro-computed tomography (micro-CT); in nine articles [7,10,13,16,18,27,29,30], the analysis was conducted through cone beam computed tomography (CBCT) [11] and computed tomography (CT) [14]. A minority of studies used other complementary analytical techniques such as fluorescence [13,22], immunohistochemistry [13], polymerase chain reaction (PCR) [19], and finite elements [16,17,18].

## 4. Discussion

This scoping review describes the biological effects of orthodontic tooth movement on the periodontium in regenerated bone defects, analysing thew bone-level response, clinical attachment level, probing pocket depth, and root integrity. These parameters are found to be influenced by multiple variables as follows: type, location, and size of the defect; graft material; timing and intensity of the force application; and total treatment duration. The size of the bone defect can be highly variable depending on the type of defect and the species analysed. Bone has an inherent ability to heal spontaneously after injury [38]. If a large defect goes beyond a certain size, which is known as a critical-size bone defect, the bone is unable to heal spontaneously [39]. For this reason, an ideal bone defect model would be one in which the defect only heals with the insertion of the graft material [40]. Only three studies [22,24,25] clearly defined how the defects that were surgically created were critical-sized and further included an “empty” or untreated blank control group to confirm the absence of spontaneous healing at the end of the study period. Ideally, a two-walled defect would be better to reproduce a true osteotomy gap, but it would be more challenging to fix the scaffold material in place [30]. Most of the included studies showed that the most frequently reproduced and studied type of defect was the extraction socket with predominantly maxillary localization. Regional variations in bone density exist between the maxilla and mandible and between the posterior and anterior regions of the same bone structure, thus making it difficult to compare studies even when they are conducted on living beings of the same species [41]. Another variable that plays an important role in the periodontal response to orthodontic tooth movement is the type of bone graft material [11,30,36,42]. The degradation of the material depends mainly on the composition of the bone substitute [43]. The current gold standard of bone grafts is the autograft since it possesses all the characteristics necessary for new bone growth, namely osteoconductivity, osteogenicity, and osteoinductivity [44]. This kind of graft also has several clinical disadvantages due to the need to remove the bone from another area causing possible complications such as pain, risk of infiltration, and scarring [45]. The increased use of alloplastic materials found in most of the studies that are included in this review is attributed to the favourable characteristics exhibited by these substitutes including biological stability, volumetric stability, osteoconductivity, degradation, absorption, and an absence of infectious risks [46]. When comparing alloplastic grafts and autografts, Hossain’s study [9] found that tricalcium phosphate exhibited a higher adaptive remodelling and biodegradative ability in response to orthodontic forces than particulate marrow and cancellous bone (PMCB). In the family of calcium phosphate bioceramics, the absorption rate is linked to the calcium-to-phosphate (Ca/P) ratio [20]. A lower Ca/P ratio of β-Tricalcium phosphate [47] in comparison with hydroxyapatite can accelerate its degradation and absorption [48]. Although alloplastics may be a viable alternative, the heterogeneity of the studies analysed led to a lack of consensus. According to Moehlhenrich [10], there is no difference between autografts, xenografts, or synthetic bone substitutes used to repair alveolar clefts, thus showing similar effects in orthodontic tooth movement and the degree of root resorption. Once the type of bone substitute was selected, the protocols of the studies analysed indicated the timing of the application of the orthodontic force. The timing ranged from immediate application after grafting surgery to six months later. Some authors suggest that tooth movement should not begin until the regenerated bone has been consolidated for 8 to 12 weeks [49,50]. Although most of the included studies began force application after four weeks to avoid an increase in the incidence of root resorption due to the lack of degradation of the bone substitute [51], some experimental studies showed that tooth movement could be applied long before the time of complete material degradation and suggested that the immediate application of force induced better periodontal regeneration by decreasing the risk of orthodontic movement inhibition [30]. Moreover, the magnitude of the applied force based on the data analysed was highly variable. This is probably due to the lack of scientific evidence concerning the level of force that may be recommended for optimal efficiency in clinical orthodontics [52]. The only observation that could be made was that heavy continuous forces increase the risks of uncontrolled tipping, increased hyalinization, and root resorption [53]. Nickel-titanium coil springs release light, continuous forces without causing rapid force decay like elastic chains do, and they do not exert high, intermittent tensile forces like stainless steel coils do [54]. After analysing the effects on the periodontium, it was observed that orthodontic tooth movement in the bone graft area promoted bone remodelling of the embedded bone, inducing resorption and subsequent deposition [19]. This remodelling depends on the multinucleated cells that play an important role in the resorption and replacement of graft materials [55]. In all the studies analysed, even though different types of materials were used, an initial phase of bone turnover in the graft zone could be observed. Moehlhenrich’s study [27] stated that autografts and xenografts exhibited a higher degree of bone formation in comparison with that observed for alloplastic substitutes. This is due to the porosity and shape of the particles that increase the surface area of xenografts, making them appropriate scaffolds for the penetration of cells mediating osteogenesis and angiogenesis [56]. However, although there was no consensus among the authors in this scoping review, most of them stated the exact opposite and attributing greater properties to the ceramic substitutes as the materials of choice, highly increasing their regenerative capabilities when conjugated with bone marrow mesenchymal stem cells (BMSCs) [12,26]. Rokn’s comparative study [55] established that there were no statistically significant differences in osteogenesis between xenogeneic and alloplastic grafts. It was more complex to determine the repercussions of orthodontic tooth movement on the clinical attachment level and the probing pocket depth because few studies dealt with these parameters. In all of them, there was an improvement in the clinical attachment level and a reduction in the probing pocket depth. This would be due to the increase in the calcium and phosphate ions that stimulate osteogenesis and cementogenesis [57]. This cementogenesis is followed by a reorganization of the periodontal ligament between the new cementum and the bone around the granules of the graft material, which occurs due to to the stimulation of the same material [9]. Dental root resorption is another important issue during biomaterial grafting in orthodontic treatment. In most of the cases analysed, root resorptions of different entities were produced from one study to another. Ru’s studies [16,17] considered root effects three-dimensionally and found that resorptions were not simply located in the areas of the highest accumulated stress but rather in those root portions subject to accumulated stress based on their surface area. Areas with a smaller surface area had more prominent root tissue craters as a result of resorption. Furthermore, it was stated that ceramic substitutes induced less root resorption than xenografts, thereby preserving the recipient area more [11,16,17]. According to some studies, the increased erosion of root cement could depend on two factors as follows: the levels of bone morphogenetic protein (BMP2) present in the alloplastic calcium phosphate graft [24,25] and the high immunogenicity of the material. Xenografts induce higher recruitment of osteoclast-like multinucleated cells promoting higher rhizolysis compared with that observed for the ceramic substitutes [58].

The included articles utilized diverse analytical methods to investigate structural changes in the periodontium resulting from orthodontic treatment. Predominantly, histomorphometry and histological analysis were employed, with Kulak CA et al. (2010) [59] considering them to be the “gold standard.” Histomorphometry quantifies newly formed bone and residual graft material percentages, while histological analysis identifies the formation of new bone matrix. Despite their appropriateness, these methods possess limitations, being time-consuming, destructive, and confined to a two-dimensional tissue evaluation [60]. Consequently, numerous studies in this scoping review [7,10,11,13,14,16,18,27,29,30] adopted three-dimensional techniques (CBCT, micro-CT, and CT). As asserted by Vandewergh et al. [61], these techniques offer insights into bone microarchitecture, which are non-destructive with excellent resolution. However, despite their advantages, three-dimensional techniques are less effective in distinguishing mineralized bone tissue from newly formed bone, thus necessitating complementary methods like histological and histomorphometric analyses [62]. Additional techniques including fluorescence, immunohistochemistry, PCR, and finite element studies were auxiliary. The former three methods helped to define biochemical processes at the cellular level influencing tooth movement [63], while the latter detailed the distribution of forces at the root level [16,17,18].

Although this scoping review includes a substantial number of papers, it has certain limitations due to the loss of several studies which did not describe the effects of such treatment on the periodontium or did not specify the type of material grafted even though they dealt with orthodontic tooth movement on grafted areas. In addition, the included studies had a high level of heterogeneity due to the different methodologies applied, with a lack of protocolization in recreating or determining the key parameters such as the species studied, type and size of the bone defect, and timing in the application of orthodontic force after grafting surgery. The studies performed on human patients especially presented a very weak design, with all of them being case reports with little scientific evidence. Even the effects on the periodontium, which are understood to be the supporting apparatus [64], were only partially studied, with the focus mainly being placed on the bone response in response to the grafted material and with most studies omitting the effects on other structures such as the gingiva, periodontal ligament, and cementum. It is mandatory to emphasize that this scoping review’s approach only maps the available evidence in a descriptive and exploratory manner rather than being analytical and explanatory like a systematic review.

Future research should be aimed at improving the design of the studies by applying a more meticulous methodology. This improvement could be achieved by treating some of the parameters evoked in the various studies with higher rigor, which include the following: the species, with it being necessary to give preference to species that are similar in their characteristics to humans by considering the size required to reproduce a bone defect of an adequate size; standardization of the size of bone defects; comparisons between the various families of grafting materials in the same study; applications of light and continuous forces; studying the long-term effects (minimum of 6 months); and a detailed study of the periodontium in its entirety with histological, histomorphometric, three-dimensional analysis methods as well as periodontal clinical examination. Studies on humans this clinical procedure is carried out, especially in branches of patients with a cleft palate and periodontal patients undergoing regenerative techniques and orthodontic treatment, should also be improved and deepened so that more scientific evidence is provided.

## 5. Conclusions

Orthodontic tooth movement in areas undergoing bone regeneration has revealed positive biological effects on periodontal health. The occurrence of increased bone formation and elevated clinical attachment levels was consistently found in almost all the studies regardless of the material used, timing, and intensity of the force applied. Although root resorption commonly arises as a complication, it does not appear to significantly impact tooth survival. Nonetheless, it is crucial to approach these findings with caution, underscoring the imperative for future preclinical and clinical studies with refined designs to generate more robust conclusions within the context of periodontal regeneration.

## Figures and Tables

**Table 1:** Search strategy for each database.

Database	Search Strategy
Web of Science	(TS=((“orthodontic movement” OR “tooth movement” OR “orthodontic treatment”))) AND TS=((“bone defect*” OR “alveolar defect*” OR “osseus defect*” OR “bone graft” OR “bone regeneration” OR “alveolar cleft”))
Scopus	(TITLE-ABS-KEY ((“orthodontic movement” OR “tooth movement” OR “orthodontic treatment”)) AND TITLE-ABS-KEY ((“bone defect*” OR “alveolar defect*” OR “osseus defect*” OR “bone graft” OR “bone regeneration” OR “alveolar cleft”)))
Embase	(“orthodontic movement” OR “tooth movement” OR “orthodontic treatment”) AND (“bone defect*” OR “alveolar defect*” OR “osseus defect*” OR “bone graft” OR “bone regeneration” OR “alveolar cleft”)
PubMed	(“orthodontic movement”[All Fields] OR “tooth movement”[All Fields] OR “orthodontic treatment”[All Fields]) AND (“bone defect*”[All Fields] OR “alveolar defect*”[All Fields] OR “osseus defect*”[All Fields] OR “bone graft”[All Fields] OR “bone regeneration”[All Fields] OR “alveolar cleft”[All Fields])
Opengrey	(orthodontic movement OR tooth movement OR orthodontic treatment) AND (bone defect OR alveolar defect OR osseus defect OR bone graft OR bone regeneration OR alveolar cleft)

**Table 2:** Main characteristics of the studies.

Authors/Year	Study Design	Species Age Gender	Type of Defect	Size Localization	Regeneration Materials
Ahn HW et al. (2014) [30]	Experimental	Dog 18 to 24 m 12 M	Extraction socket	- 5 mm (mesio-distal) × 5 mm (buco-lingual) × 7 mm (vestibular) - Mx FPM	DBB (Bio-Oss) and DBM (OrthoBlast II)
Araújo M et al. (2001) [34]	Experimental	Dog 1 y 5 N/R	Extraction socket	- N/R - Mb FPM, SPM, FoPM	DBB (BioOssA)
Attia MS et al. (2012) [36]	Experimental	Human 25 to 48 y 10 F, 5 M	Infrabony defects	- PPD 5 > mm - 45 sites involved not specified	BG (Bio-Glass)
Cardaropoli D et al. (2006) [32]	Case report	Human N/R 3 M	Infrabony defects	- PPD > 6 mm - Mx CI	DBB (Bio-Oss)
Fung K et al. (2012) [37]	Case report	Human 68 y 1 F	Infrabony defects	- 15 mm height - Mx CI	EMD (Emdogain) and BCP
Hossain M et al. (1996) [9]	Experimental	Dog 1 y 9 N/R	Extraction socket	- N/R - Mx SI/TI	AB and β-TCP
Jiang S et al. (2020) [11]	Experimental	Dog 1 y 9 M	Extraction socket	- 4.5 mm diameter × 6 mm deep - Mx FPM	BioCaP and DBB
Kawamoto T et al. (2003) [24]	Experimental	Dog 1.6 to 2.6 y 8 F	Extraction socket	- 5 mm diameter - Mx FPM	rhBMP-2 with PGS
Kawamoto T et al. (2002) [25]	Experimental	Dog 1 y 5 m to 2 y 3 m 8 F	Extraction socket	- 5 mm diameter - Mx SPM	rhBMP-2with PGS
Klein Y et al. (2019) [29]	Experimental	Mouse 6/7 w 44 M	Extraction socket	- N/R - Mx FM	BB
Klein Y et al. (2020) [7]	Experimental	Mouse 6/7 w 54 M	Extraction socket	- N/R - Mx FM	AG and β-TCP
Lee KB et al. (2014) [31]	Experimental	Dog 1/2 y 6 M	Periodontal defects	- N/R - Mx and Mb buccal bone surface	DBB, (Bio-Oss), IB, SB, BCP
Li YH et al. (2018) [12]	Experimental	Rabbit 5 to 6 m 30 N/R	Extraction socket	- 6 mm × 4 mm × 8 mm - Mb FM	BMSCs and β-TCP
Ma Z et al. (2021) [13]	Experimental	Dog 1.5 y 6 M	Dehiscencetype defects	- 5 mm width, 6 mm height - Distal root of Mx SPM	DBB (Bio-Oss)
Machibya FM et al. (2018) [14]	Experimental	Dog 18 m 6 M	Extraction socket	- 5 mm deep, 7 mm long (mesial-distal) and 5 mm wide (buccolingual) - Mx and Mb FPM	DBB (Bio-Oss) and β-TCP
Mao L et al. (2013) [15]	Observational	Human 18.3 ± 4.2 y 30 N/R	Unilateral cleft lip and palate	- N/R - Mx C on the cleft side	AB
Moehlhenrich SC et al. (2021) [27]	Experimental	Rat 8 w 21 M	Alveolar cleft	- 1.7 mm diameter - Between Mx FM and Mx anterior part	AB, XHB, β-TCP, and HA
Moehlhenrich SC et al. (2022) [10]	Experimental	Rat 8 w 21 M	Alveolar cleft	- N/R - Between Mx FM and Mx anterior part	AB, XHB, β-TCP, and HA
Oltramari PVP et al. (2007) [33]	Experimental	Minipig 12 m 6 M	Extraction socket	- N/R - Mx and Mb mesial aspect of FM	DBB, BMP, and HA
Reichert C et al. (2011) [28]	Case report	Human 11.6 y, 13.10 y, 11.2 y 1 F, 2 M	Extraction socket	- N/R - Mx SPM - Mx FPM - Mb FPM	NanoBone
Ru N et al. (August 2016) [18]	Experimental	Rat 5 w 60 M	Extraction socket	- 3 mm × 2 mm × 2 mm - Mx FM	BCP (bone ceramic), DBB(BioOss)
Ru N et al. (April 2016) [17]	Experimental	Rat 5 w 60 M	Extraction socket	- 3 mm × 2 mm × 2 mm - Mx FM	BCP (bone ceramic); DBB(BioOss)
Ru N et al. (2018) [16]	Experimental	Rat 5 w 60 M	Extraction socket	- 3 mm × 2 mm × 2 mm - Mx FM	BCP (bone ceramic); DBB(BioOss)
Sun J et al. (2018) [19]	Experimental	Rat 8 w 39 M	Alveolar cleft	- N/R - Mx FM	AB
Tanimoto K et al. (2015) [26]	Experimental	Dog 3 m 3 F	Alveolar cleft	- 5 mm width × 10 mm length - Mx TI	BMSCs and HA
Wang L Lei et al. (2017) [20]	Experimental	Dog 1.5 y 2 M	Alveolar bone defect	- 4 mm high × 3 mm wide × 3 mm deep - Mx and Mb TI	NBCP
Yilmaz S et al. (2000) [35]	Case report	Human 16 y 1 M	Unilateral cleft and palate	- N/R - Mx anterior region	DFDBA and BG
Zhang D et al. (2011) [21]	Experimental	Dog 24 w 7 M	Alveolar cleft	- 10 × 5 × 15 mm - Mx TI	BMSCs and β-TCP, β-TCP, AB
Zhang FF et al. (2019) [22]	Experimental	Rabbit 20 to 24 y 40 N/R	Extraction socket	- 6 mm × 4 mm × 8 mm - Mb FM	BMSCs and β-TCP
Zhou J et al. (2018) [23]	Case report	Human 38.4 y 7 F, 2 M	Vertical bone defect	- More than one-third of the root length - Mx I	DBB (Bio-Oss)

Abbreviations: w, weeks; m, months; y, years; M, male; F, female; Mx, maxillar; Mb, mandibular; FPM, first premolar; SPM, second premolar; TPM, third premolar; FoPM, fourth premolar; FM, first molar; C, canine; TI, third incisor; I, incisor; DBB, deproteinized bovine bone; DBM, demineralized bone matrix; AB, autogenic bone; BB, bovine bone; AG, allograft; IB, irradiated bone; SB, synthetic bone; XHB, xenogenic human bone; BMP, bone morphogenetic protein; EDM, enamel matrix derivative; NBCP, nano-biphasic calcium phosphate; rhBMP-2; recombinant human morphogenetic protein-2; PGS, gelatin sponge complex; DFDBA, demineralized freeze-dried cortical bone allograft; β-TCP, β-tricalcium phosphate; BG, bioactive glass; NanoBone, nanoparticle-hydroxyapatite; BCP, biphasic calcium phosphate; HA, hydroxyapatite; BioCaP, BMP2-functionalized biomimetic calcium phosphate; BMSCs, bone marrow stem cells; N/R, not reported.

**Table 3:** Type of samples and their characteristics.

	Dog	Human	Rat	Mouse	Rabbit	Minipig
Number of studies	12	7	6	2	2	1
Sample (n)	84	62	261	98	70	6
Male	51	13	261	98	0	6
Female	15	19	0	0	0	0
N/R	18	30	0	0	70	0
Age	3–27 months	11.2–68 years	5–12 weeks	6–7 weeks	20–24 weeks	12 months

**Table 4:** Parameters of orthodontic tooth movement.

Authors/Year	Localization of OTM	Time after Surgery/Treatment	Characteristics of the Force	Total Duration of OTM	Amount of OTM
Ahn HW et al. (2014) [30]	Between Mx-C and Mx-SPM	- Immediately - 2 w - 12 w	- Mesial - 100 g - NiTi closed coil spring	- 6 w	1.75 to 3.44 mm
Araújo M et al. (2001) [34]	Between Mb TPM and Mb FM	- 3 m	- Distal - 30 to 50 cN - Closed coil spring	- 2/4 w	3.85 ± 57 mm
Attia MS et al. (2012) [36]	N/R	- Immediately - 2 m	- N/R - 10 to 15 g - SS segmented arch	- 12 m	N/R
Cardaropoli D et al. (2006) [32]	Mx CI	- 2 w	- Mesial - N/R - Segmented technique—edgewise utility arch	- 6 m - 9 m - 4 m	N/R
Fung K et al. (2012) [37]	Between Mx CI	- 1 w after surgery	- Apical - 40 g - 014″ NiTi overlay with 017″ × 025″ SS base archwire	- 2 m	1 mm
Hossain M et al. (1996) [9]	Between Mx C and CI	- 2 to 4 w	- Distal - N/R - Coil spring	- 9 to 15 w	N/R
Jiang S et al. (2020) [11]	Between Mx SPM and C	- N/R	- Mesial - 150 g - NiTi coil spring	- 8 w	DBB group: 3.59 ± 1.25 BioCap group: 2.90 ± 0.84
Kawamoto T et al. (2003) [24]	Between Max SPM and C	- 4 m	- Mesial - 100 g - NiTi coil spring	- 2 m	2 mm
Kawamoto T et al. (2002) [25]	Max 2PM	- 4 m	- Mesial - 100 g - NiTi closed coil spring	- 2 m	2 mm
Klein Y et al. (2019) [29]	Between Mx SM and I	- 4 w	- Mesial - 10 g - NiTi closed coil spring	- 2–3 w	550.36 μm ± 101.52
Klein Y et al. (2020) [7]	Between Mx SM and I	- 4 w	- Mesial - 10 g - NiTi closed coil spring	- 3 w	β-TCP group: 707.3 ± 30.6 μm AG group 648.3 ± 31.6 μm
Lee KB et al. (2014) [31]	Between Mx SPM and TPM and Mb SPM and TPM	- Immediately	- Buccal tipping - 200 g - Closed coil spring	- 6 w	DBBM group: 20.81 ± 8.07° IB group: 16.08 ± 4.14° SB group: 27.26 ± 7.27°
Li YH et al. (2018) [12]	Between Mx I and SM	- Immediately	- Mesial - 80 g - NiTi tension spring	- 4 w	BMSCs + β-TCP group: 3.17 ± 0.26 β-TCP group: 2.79 ± 0.12
Ma Z et al. (2021) [13]	Between Mx C and FPM	- Immediately - 4 w - 8 w	- Buccal - 50 g - NiTi closed coil spring	- 8 w	Expansion and buccal tipping: Immediately force application group: 2.42 mm and 9.03 ± 1.02 4 w after surgery force application group: 1.25 mm and 5.32 ± 2.19 8 w after surgery force applicationgroup: 1.62 mm and 3.24 ± 1.27
Machibya FM et al. (2018) [14]	Between Mx and MbC and SPM	- 1 m - 2 m	- Mesial - 150 g - NiTi closed coil spring	- 7–8 w	Bio-Oss group 4.22 mm β-TCP group: 4.76 mm
Mao L et al. (2013) [15]	Between Mc C and CI	- -When the canines were levelled and had moved labially	- Mesial - N/R - MBT bracket system, 0.022 × 0.028 inches	- N/R	N/R
Moehlhenrich SC et al. (2021) [27]	Between Mx FM and I	- 4 w	- Mesial - 0.14 N - Niti closed coil spring	- 8 w	N/R
Moehlhenrich SC et al. (2022) [10]	Between Mx FM and I	- 4 w	- Mesial - 0.14 N - NiTi closed coil spring	- 8 w	SB group: 0.82 ± 0.72 mm XHB group: 0.78 ± 0.69 mm AB group: 0.67 ± 0.27 mm
Oltramari PVP et al. (2007) [33]	Between Mx and Mb FM and DTM	- 3 m	- Mesial - 4.5 N - Niti closed coil spring	- N/R	4 mm
Reichert C et al. (2011) [28]	Between Mx SPM, between Mx FPM, between Mb FPM	- 6 w	- N/R - 200 g - NiTi closed coil spring	- 7/8 m - 6 m - 7 m	N/R
Ru N et al. (August 2016) [18]	Between Mx SM and I	- 4 w	- Mesial - 10 g - NiTi closed coil spring	- 28 d	BCP with a lower amount of OTM than DBB
Ru N et al. (April 2016) [17]	Between Mx SM and I	- 4 w	- Mesial - 10 g - NiTi closed coil spring	- 28 d	BCP with a lower amount of OTM than DBB
Ru N et al. (2018) [16]	Between Mx SM and I	- 4 w	- Mesial - 10 g - NiTi closed coil spring	- 28 d	BCP with a lower amount of OTM than DBB
Sun J et al. (2018) [19]	Between Mx SM and I	- 8 w	- Mesial - 100 g - Tension spring	- 5 d	N/R
Tanimoto K et al. (2015) [26]	Between Mx SI and C	- 6 m	- Distal and mesial - 100 g - Elastic chain	- 6 m	6 mm
Wang L Lei et al. (2017) [20]	Between Mx and Mb TI and C	- 24 w	- Labial - 100 g - Australian wire of 0.016 inches	- 2 w	N/R
Yilmaz S et al. (2000) [35]	Between Mx LI and C	- 6 m	- Mesial tip - N/R - Uprighting spring and Z band	- 32 m	N/R
Zhang D et al. (2011) [21]	Between Mx LI and C	- 8 w	- Distal - 50 g - NiTi closed coil spring	- 12 w	bMSCs/β-TCP group: 5.345 ± 0.936 mm β-TCP group: 6.986 ± 1.412 mm AB group: 4.665 ± 0.483 mm
Zhang FF et al. (2019) [22]	Between Mb SM and FPM	- 2 w - 4 w - 8 w - 12 w	- Mesial - 80 g - NiTi tension spring	4 w	- 0.97 ± 0.18 mm at 2 w - 1.03 ± 0.15 mm at 4 w - 1.69 ± 0.16 mm at 8 w - 1.11 ± 01.17 mm at 12 w
Zhou J et al. (2018) [23]	Mx I	- 3 m	- Apical - 15 g - Segmented arch	11.3 m	N/R

Abbreviations: Mx, maxillar; Mb, mandibular; FPM, first premolar; SPM, second premolar; TPM, third premolar; FM, first molar; SM, second molar; CI, central incisor; LI, lateral incisor; I, incisor; C, canine; w, weeks; m, months; NiTi, nickel titanium; SS, stainless steel; N, newton; OTM, orthodontic tooth movement; β-TCP, β-tricalcium phosphate; AB, autogenic bone; AG, allograft; BCP, biphasic calcium phosphate; DBB, deproteinized bovine bone; XHB, xenogenic human bone; SB, synthetic bone; BMSCs, bone marrow stem cells; N/R, Not reported.

**Table 5:** Biological repercussions on the periodontium complex.

Authors/Year	Bone Formation/Resorption	Clinical Attachment Level	Roots Integrity/Resorption	Probing Pocket Depth	Methods of Analysis
Ahn HW et al. (2014) [30]	Increased BF	N/R	N/R	N/R	- Histo - Micro CT
Araújo M et al. (2001) [34]	Increased BF	N/R	Minor RR	N/R	- Histo
Attia MS et al. (2012) [36]	Increased BF	Increased clinical attachment	N/R	N/R	- Clinical measures - Rx
Cardaropoli D et al. (2006) [32]	Increased BF	Increased CAL	N/R	Decreased PPD	- Clinical measures - Rx
Fung K et al. (2012) [37]	Increased BF	Increased CAL	No RR	Decreased PPD	- Clinical measures - Rx
Hossain M et al. (1996) [9]	Increased BF	Increased attachments of the PDL fibers	Minor RR	N/R	- Histo - Rx
Jiang S et al. (2020) [11]	Increased BF	N/R	Increased RR in BioCap	Increased PPD	- CBCT - Clinical measures - Histo
Kawamoto T et al. (2003) [24]	Increased BF	N/R	Partial cementum resorption	N/R	- Histo - Histom
Kawamoto T et al. (2002) [25]	Increased BF	N/R	Negligible cementum resorption	N/R	- Histo - Histom
Klein Y et al. (2019) [29]	Increased BF	N/R	N/R	N/R	- Histo - Micro CT
Klein Y et al. (2020) [7]	Increased BF	N/R	N/R	N/R	- Histo - Micro CT
Lee KB et al. (2014) [31]	Increased BF	N/R	Partial cementum resorption	Increased PD	- Clinical measures - Histo
Li YH et al. (2018) [12]	Increased BF (better in BMSCs +β-TCP than β-TCP)	N/R	No RR	N/R	- Histo
Ma Z et al. (2021) [13]	Increased BF	N/R	N/R	N/R	- Fm - Ic - Histo - Histom - Micro CT
Machibya FM et al. (2018) [14]	Increased BF	N/R	N/R	N/R	- Clinical measures - CT
Mao L et al. (2013) [15]	Decreased BF in less than 25% of the sample	N/R	Slight RR	N/R	- Clinical measures - Rx
Moehlhenrich SC et al. (2021) [27]	Increased BF (highest in the XHB group and lowest in the SB group)	N/R	N/R	N/R	- Histo - Micro CT
Moehlhenrich SC et al. (2022) [10]	N/R	N/R	RR in all groups	N/R	- Histo - Micro CT
Oltramari PVP et al. (2007) [33]	BR and BF were balanced	N/R	Slight RR	N/R	- Histo - Histom
Reichert C et al. (2011) [28]	N/R	N/R	No RR	N/R	- Clinical measures - Rx
Ru N et al. (August 2016) [18]	BCP with more BF than DBB	N/R	N/R	N/R	- FE - Micro CT - Ni
Ru N et al. (April 2016) [17]	BCP with more BF than DBB	N/R	BCP with less RR than DBB	N/R	- FE - Micro CT - Ni
Ru N et al. (2018) [16]	BCP with more BF than DBB	N/R	BCP with less RR than DBB	N/R	- CT - FE - Histo - Micro - Ni
Sun J et al. (2018) [19]	Increased BF	N/R	N/R	N/R	- Histo - PCR
Tanimoto K et al. (2015) [26]	Increased BF	N/R	No root resorption	N/R	- Histo - Rx
Wang L Lei et al. (2017) [20]	No difference between newly formed periodontium and normal periodontal tissues	No difference between newly formed periodontium and normal periodontal tissues	No difference between newly formed periodontium and normal periodontal tissues	No difference between newly formed periodontium and normal periodontal tissues	- Histo
Yilmaz S et al. (2000) [35]	Increased BF	No gums recessions	No RR	N/R	- Clinical measures - Rx
Zhang D et al. (2011) [21]	Increased BF (higher in BMSCs/β-TCP group than β-TCP group)	N/R	N/R	N/R	- Fm - Histo - Rx
Zhang FF et al. (2019) [22]	N/R	N/R	N/R	N/R	- Histo
Zhou J et al. (2018) [23]	Increased BF	Increased CAL	N/R	Decreased PPD	- Clinical measures - Rx

Abbreviations: BF, bone formation; BR, bone resorption; CAL, clinical attachment level; PPD, probing pocket depth; RR, root resorption; β-TCP, β-tricalcium phosphate; BCP, biphasic calcium phosphate; DBB, deproteinized bovine bone; SB, synthetic bone; XHB, xenogenic human bone; BMSCs, bone marrow stem cells; histo, histology; histom, histomorphometry; Rx examination, radiographic examination; microCT, microcomputed tomography; CT, computed tomography; CBCT, cone beam computed tomography; fm, fluorescence microscopy; ic, immunohistochemistry; FE, finite element; ni, nanoindentation; PCR, polymerase chain reaction; N/R, not reported.

## Data Availability

The data presented in this study are available in the article.

## References

[1] Zhao R., Yang R., Cooper P.R., Khurshid Z., Shavandi A., Ratnayake J. (2021). Bone Grafts and Substitutes in Dentistry: A Review of Current Trends and Developments. Molecules.

[2] Kolk A., Handschel J., Drescher W., Rothamel D., Kloss F., Blessmann M., Heiland M., Wolff K.-D., Smeets R. (2012). Current Trends and Future Perspectives of Bone Substitute Materials—From Space Holders to Innovative Biomaterials. J. Cranio-Maxillofac. Surg..

[3] Lu J., Wang Z., Zhang H., Xu W., Zhang C., Yang Y., Zheng X., Xu J. (2022). Bone Graft Materials for Alveolar Bone Defects in Orthodontic Tooth Movement. Tissue Eng. Part B Rev..

[4] Sendyk M., Linhares D.S., Pannuti C.M., de Paiva J.B., Neto J.R. (2019). Effect of Orthodontic Treatment on Alveolar Bone Thickness in Adults: A Systematic Review. Dent. Press J. Orthod..

[5] Liu Y., Li C.X., Nie J., Mi C.B., Li Y.M. (2023). Interactions between Orthodontic Treatment and Gingival Tissue. Chin. J. Dent. Res..

[6] Yassir Y.A., McIntyre G.T., Bearn D.R. (2021). Orthodontic Treatment and Root Resorption: An Overview of Systematic Reviews. Eur. J. Orthod..

[7] Klein Y., Kunthawong N., Fleissig O., Casap N., Polak D., Chaushu S. (2020). The Impact of Alloplast and Allograft on Bone Homeostasis: Orthodontic Tooth Movement into Regenerated Bone. J. Periodontol..

[8] Tricco A.C., Lillie E., Zarin W., O’Brien K.K., Colquhoun H., Levac D., Moher D., Peters M.D.J., Horsley T., Weeks L. (2018). PRISMA Extension for Scoping Reviews (PRISMA-ScR): Checklist and Explanation. Ann. Intern. Med..

[9] Hossain M.Z., Kyomen S., Tanne K. (1996). Biologic Responses of Autogenous Bone and Beta-Tricalcium Phosphate Ceramics Transplanted into Bone Defects to Orthodontic Forces. Cleft Palate-Craniofac. J..

[10] Möhlhenrich S.C., Kniha K., Magnuska Z., Chhatwani S., Hermanns-Sachweh B., Gremse F., Hölzle F., Danesh G., Modabber A. (2022). Development of Root Resorption during Orthodontic Tooth Movement after Cleft Repair Using Different Grafting Materials in Rats. Clin. Oral Investig..

[11] Jiang S., Liu T., Wu G., Li W., Feng X., Pathak J.L., Shi J. (2020). BMP2-Functionalized Biomimetic Calcium Phosphate Graft Promotes Alveolar Defect Healing during Orthodontic Tooth Movement in Beagle Dogs. Front. Bioeng. Biotechnol..

[12] Li Y.-H., Zhang F.-F., Bao S.-J., Wei B., Gong Y. (2018). Study on Periodontal Responses on the Compression Side during Early Tooth Movement into Alveolar Defect Regenerated by a Tissue Engineering Bone. Shanghai Kou Qiang Yi Xue Shanghai J. Stomatol..

[13] Ma Z., Wang Z., Zheng J., Chen X., Xu W., Zou D., Zhang S., Yang C. (2021). Timing of Force Application on Buccal Tooth Movement into Bone-Grafted Alveolar Defects: A Pilot Study in Dogs. Am. J. Orthod. Dentofac. Orthop..

[14] Machibya F.M., Zhuang Y., Guo W., You D., Lin S., Wu D., Chen J. (2018). Effects of Bone Regeneration Materials and Tooth Movement Timing on Canine Experimental Orthodontic Treatment. Angle Orthod..

[15] Mao L.-X., Shen G.-F., Fang B., Xia Y.-H., Ma X.-H., Wang B. (2013). Bone Grafting, Corticotomy, and Orthodontics: Treatment of Cleft Alveolus in a Chinese Cohort. Cleft Palate-Craniofac. J..

[16] Ru N., Liu S.S.-Y., Bai Y., Li S., Liu Y., Zhou G. (2018). Microarchitecture and Biomechanical Evaluation of BoneCeramic Grafted Alveolar Defects during Tooth Movement in Rat. Cleft Palate-Craniofac. J. Off. Publ. Am. Cleft Palate-Craniofac. Assoc..

[17] Ru N., Liu S.S.-Y., Bai Y., Li S., Liu Y., Wei X. (2016). BoneCeramic Graft Regenerates Alveolar Defects but Slows Orthodontic Tooth Movement with Less Root Resorption. Am. J. Orthod. Dentofac. Orthop..

[18] Ru N., Liu S.S.-Y., Bai Y., Li S., Liu Y., Zhou G. (2016). In Vivo Micro–Computed Tomography Evaluation of BoneCeramic Grafted Alveolar Defects during Orthodontic Tooth Movement. Angle Orthod..

[19] Sun J., Zhang X., Li R., Chen Z., Huang Y., Chen Z. (2018). Biological Effects of Orthodontic Tooth Movement into the Grafted Alveolar Cleft. J. Oral Maxillofac. Surg..

[20] Wang L., Hou H., Yu S., Guan A., Liao Y. (2017). The Histological Study of Orthodontic Force on the Periodontal Tissues Regenerated by Nano Bioceramics in Beagle Dogs. Proceedings of the 2nd International Conference on Biomedical and Biological Engineering 2017 (BBE 2017).

[21] Zhang D., Chu F., Yang Y., Xia L., Zeng D., Uludağ H., Zhang X., Qian Y., Jiang X. (2011). Orthodontic Tooth Movement in Alveolar Cleft Repaired with a Tissue Engineering Bone: An Experimental Study in Dogs. Tissue Eng. Part A.

[22] Zhang F.-F., Bao S.-J., Ye S.-J., Wei B., Gong Y. (2019). Study of the Timing of Tooth Movement after Repair of Alveolar Bone Defects by Rabbit BMSCs Combined with Beta-TCP. Shanghai Kou Qiang Yi Xue Shanghai J. Stomatol..

[23] Zhou J., Shu R., Gong Y., Xie Y. (2018). Therapeutic Effect of Orthodontic Intrusion Combined with Periodontal Regenerative Surgery in the Treatment of Pathologic Migration of Upper Incisors. J. Shanghai Jiaotong Univ. (Med. Sci.).

[24] Kawamoto T., Motohashi N., Kitamura A., Baba Y., Suzuki S., Kuroda T. (2003). Experimental Tooth Movement into Bone Induced by Recombinant Human Bone Morphogenetic Protein-2. Cleft Palate-Craniofac. J..

[25] Kawamoto T., Motohashi N., Kitamura A., Baba Y., Takahashi K., Suzuki S., Kuroda T. (2002). A Histological Study on Experimental Tooth Movement into Bone Induced by Recombinant Human Bone Morphogenetic Protein-2 in Beagle Dogs. Cleft Palate-Craniofac. J..

[26] Tanimoto K., Sumi K., Yoshioka M., Oki N., Tanne Y., Awada T., Kato Y., Sugiyama M., Tanne K. (2015). Experimental Tooth Movement into New Bone Area Regenerated by Use of Bone Marrow–Derived Mesenchymal Stem Cells. Cleft Palate-Craniofac. J..

[27] Möhlhenrich S.C., Kniha K., Magnuska Z., Hermanns-Sachweh B., Gremse F., Hölzle F., Danesh G., Modabber A. (2021). Evaluation of Different Grafting Materials for Alveolar Cleft Repair in the Context of Orthodontic Tooth Movement in Rats. Sci. Rep..

[28] Reichert C., Wenghöfer M., Götz W., Jäger A. (2011). Pilot Study on Orthodontic Space Closure after Guided Bone Regeneration. J. Orofac. Orthop. Fortschritte Kieferorthopädie.

[29] Klein Y., Fleissig O., Stabholz A., Chaushu S., Polak D. (2019). Bone Regeneration with Bovine Bone Impairs Orthodontic Tooth Movement despite Proper Osseous Wound Healing in a Novel Mouse Model. J. Periodontol..

[30] Ahn H.-W., Ohe J.-Y., Lee S.-H., Park Y.-G., Kim S.-J. (2014). Timing of Force Application Affects the Rate of Tooth Movement into Surgical Alveolar Defects with Grafts in Beagles. Am. J. Orthod. Dentofac. Orthop..

[31] Lee K.-B., Lee D.-Y., Ahn H.-W., Kim S.-H., Kim E.-C., Roitman I. (2014). Tooth Movement out of the Bony Wall Using Augmented Corticotomy with Nonautogenous Graft Materials for Bone Regeneration. BioMed Res. Int..

[32] Cardaropoli D., Re S., Manuzzi W., Gaveglio L., Cardaropoli G. (2006). Bio-Oss Collagen and Orthodontic Movement for the Treatment of Infrabony Defects in the Esthetic Zone. Int. J. Periodont. Restor. Dent..

[33] Oltramari P.V.P., Navarro R.d.L., Henriques J.F.C., Taga R., Cestari T.M., Ceolin D.S., Janson G., Granjeiro J.M. (2007). Orthodontic Movement in Bone Defects Filled with Xenogenic Graft: An Experimental Study in Minipigs. Am. J. Orthod. Dentofac. Orthop..

[34] Araujo M.G., Carmagnola D., Berglundh T., Thilander B., Lindhe J. (2001). Orthodontic Movement in Bone Defects Augmented with Bio-OssR^®^: An Experimental Study in Dogs. J. Clin. Periodontol..

[35] Yılmaza S., Kılıçb A.R., Kelesc A., Efeoğlud E. (2000). Reconstruction of an Alveolar Cleft for Orthodontic Tooth Movement. Am. J. Orthod. Dentofac. Orthop..

[36] Attia M.S., Shoreibah E.A., Ibrahim S.A., Nassar H.A. (2012). Regenerative Therapy of Osseous Defects Combined with Orthodontic Tooth Movement. J. Int. Acad. Periodontol..

[37] Fung K., Chandhoke T.K., Uribe F., Schincaglia G.P. (2012). Periodontal Regeneration and Orthodontic Intrusion of a Pathologically Migrated Central Incisor Adjacent to an Infrabony Defect. J. Clin. Orthod..

[38] Giannoudis P.V., Jones E., Einhorn T.A. (2011). Fracture Healing and Bone Repair. Injury.

[39] Perry C.R. (1999). Bone Repair Techniques, Bone Graft, and Bone Graft Substitutes. Clin. Orthop. Relat. Res..

[40] Van der Stok J., Van Lieshout E.M.M., El-Massoudi Y., Van Kralingen G.H., Patka P. (2011). Bone Substitutes in the Netherlands—A Systematic Literature Review. Acta Biomater..

[41] Muschler G.F., Raut V.P., Patterson T.E., Wenke J.C., Hollinger J.O. (2010). The Design and Use of Animal Models for Translational Research in Bone Tissue Engineering and Regenerative Medicine. Tissue Eng. Part B Rev..

[42] Hara Y., Murakami T., Kajiyama K., Maeda K., Akamine A., Nagamine N., Miyatake S., Abe T., Azemoto Y., Aono M. (1989). Application of calcium phosphate ceramics to periodontal therapy. 8. Effects of orthodontic force on repaired bone with hydroxyapatite. Nihon Shishubyo Gakkai Kaishi.

[43] Qu H., Fu H., Han Z., Sun Y. (2019). Biomaterials for Bone Tissue Engineering Scaffolds: A Review. RSC Adv..

[44] Laurencin C., Khan Y., El-Amin S.F. (2006). Bone Graft Substitutes. Expert Rev. Med. Devices.

[45] Szabó G., Huys L., Coulthard P., Maiorana C., Garagiola U., Barabás J., Németh Z., Hrabák K., Suba Z. (2005). A Prospective Multicenter Randomized Clinical Trial of Autogenous Bone versus Beta-Tricalcium Phosphate Graft Alone for Bilateral Sinus Elevation: Histologic and Histomorphometric Evaluation. Int. J. Oral Maxillofac. Implant..

[46] Hsu Y.-T., Wang H.-L. (2013). How to Select Replacement Grafts for Various Periodontal and Implant Indications. Clin. Adv. Periodont..

[47] Alizadeh-Osgouei M., Li Y., Wen C. (2019). A Comprehensive Review of Biodegradable Synthetic Polymer-Ceramic Composites and Their Manufacture for Biomedical Applications. Bioact. Mater..

[48] Yu X., Tang X., Gohil S.V., Laurencin C.T. (2015). Biomaterials for Bone Regenerative Engineering. Adv. Healthc. Mater..

[49] Cope J.B., Samchukov M.L. (2000). Regenerate Bone Formation and Remodeling during Mandibular Osteodistraction. Angle Orthod..

[50] Uckan S., Guler N., Arman A., Mutlu N. (2006). Mandibular Midline Distraction Using a Simple Device. Oral Surg. Oral Med. Oral Pathol. Oral Radiol. Endod..

[51] Cottrell D.A., Wolford L.M. (1998). Long-Term Evaluation of the Use of Coralline Hydroxyapatite in Orthognathic Surgery. J. Oral Maxillofac. Surg..

[52] Ren Y., Maltha J.C., Kuijpers-Jagtman A.M. (2003). Optimum Force Magnitude for Orthodontic Tooth Movement: A Systematic Literature Review. Angle Orthod..

[53] Li Y., Zhan Q., Bao M., Yi J., Li Y. (2021). Biomechanical and Biological Responses of Periodontium in Orthodontic Tooth Movement: Up-Date in a New Decade. Int. J. Oral Sci..

[54] Bauer T.W., Muschler G.F. (2000). Bone Graft Materials. An Overview of the Basic Science. Clin. Orthop. Relat. Res..

[55] Rokn A.R., Khodadoostan M.A., Ghahroudi A.A.R.R., Motahhary P., Fard M.J.K., Bruyn H.D., Afzalifar R., Soolar E., Soolari A. (2011). Bone Formation with Two Types of Grafting Materials: A Histologic and Histomorphometric Study. Open Dent. J..

[56] Artzi Z., Tal H., Dayan D. (2000). Porous Bovine Bone Mineral in Healing of Human Extraction Sockets. Part 1: Histomorphometric Evaluations at 9 Months. J. Periodontol..

[57] Cate R.T. (1998). Oral Histology: Development, Structure and Function.

[58] Liu T., Zheng Y., Wu G., Wismeijer D., Pathak J.L., Liu Y. (2017). BMP2-Coprecipitated Calcium Phosphate Granules Enhance Osteoinductivity of Deproteinized Bovine Bone, and Bone Formation during Critical-Sized Bone Defect Healing. Sci. Rep..

[59] Kulak C.A., Dempster D.W. (2010). Bone histomorphometry: A concise review for endocrinologists and clinicians. Arq. Bras. Endocrinol. Metabol..

[60] Rentsch C., Schneiders W., Manthey S., Rentsch B., Rammelt S. (2014). Comprehensive histological evaluation of bone implants. Biomatter.

[61] Vandeweghe S., Coelho P.G., Vanhove C., Wennerberg A., Jimbo R. (2013). Utilizing micro-computed tomography to evaluate bone structure surrounding dental implants: A comparison with histomorphometry. J. Biomed. Mater. Res. B Appl. Biomater..

[62] Shanbhag S., Suliman S., Pandis N., Stavropoulos A., Sanz M., Mustafa K. (2019). Cell therapy for orofacial bone regeneration: A systematic review and meta-analysis. J. Clin. Periodontol..

[63] Ren Y., Vissink A. (2008). Cytokines in crevicular fluid and orthodontic tooth movement. Eur. J. Oral Sci..

[64] Fiorellini J.P., Kao D.W., Kim D.M., Uzel N.G. (2015). Anatomy of the Periodontium. Carranza’s Clinical Periodontology.

